# P-1312. Exploring Combination Regimens Involving Cefidericol for the Treatment of Stenotrophomonas maltophilia Infections

**DOI:** 10.1093/ofid/ofaf695.1500

**Published:** 2026-01-11

**Authors:** Hashem Haj Ebrahimi, Hiyam Ghneim

**Affiliations:** University of Debrecen, Debrecen, Hajdu-Bihar, Hungary; University of Debrecen, Debrecen, Hajdu-Bihar, Hungary

## Abstract

**Background:**

*Stenotrophomonas maltophilia* (SM) is a multidrug-resistant (MDR), opportunistic pathogen that affects immunocompromised and critically ill patients. It causes respiratory and bloodstream infections and exhibits intrinsic resistance to many antibiotics. There are no FDA-approved drugs for SM, and rising resistance to trimethoprim-sulfamethoxazole (TMP-SMX) has complicated treatment. Cefiderocol (CFDC) is a promising alternative. This study investigates the clinical outcomes of combination therapies involving CFDC for treatment of SM infections.

**Figure 1:**
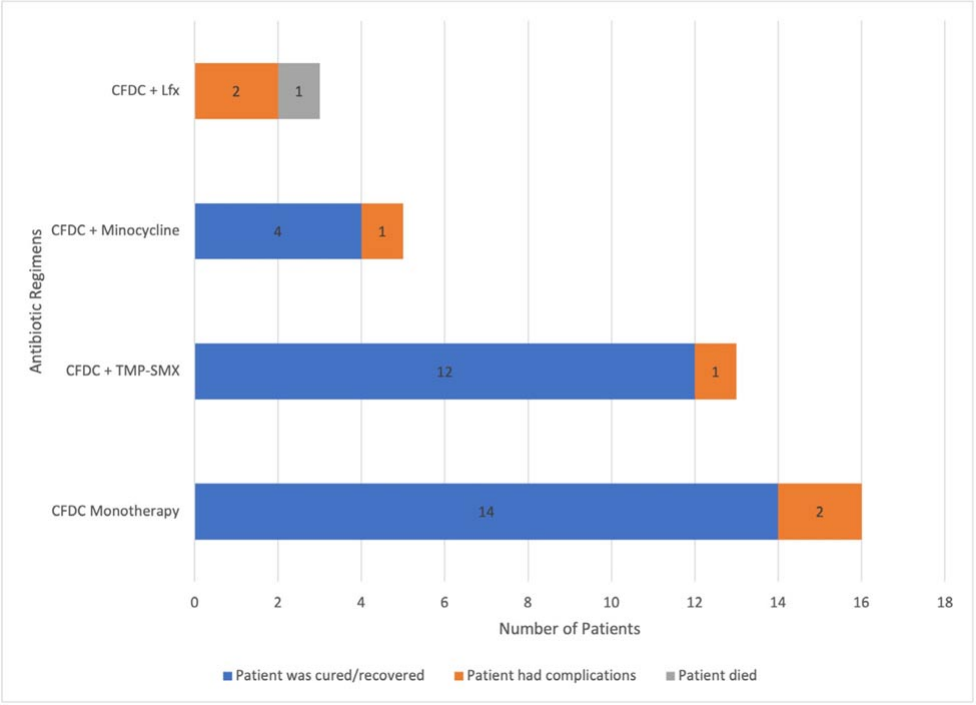
Cefiderocol Antibiotic Combinations Used in Treatment Regimens with S. maltophilia Infection

**Figure 2:**
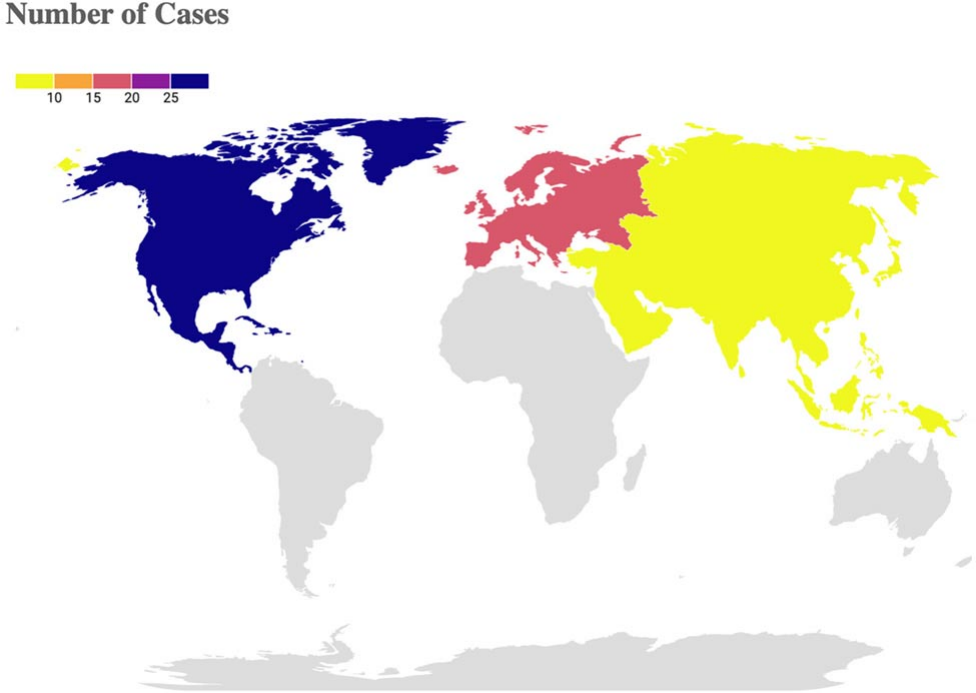
Geographic Distribution of S. Maltophilia Cases

**Methods:**

SM cases treated with CFDC were identified from PubMed and Embase databases (2020-2024) using relevant MeSH and Entree terms. Articles were screened through Rayyan.ai following PRISMA guidelines. From 186 articles, 53 met the inclusion criteria. Data was analyzed, variables included gender, geography, antibiotic combinations, resistance patterns, and clinical outcomes. Descriptive statistical analysis was performed.

**Figure 3:**
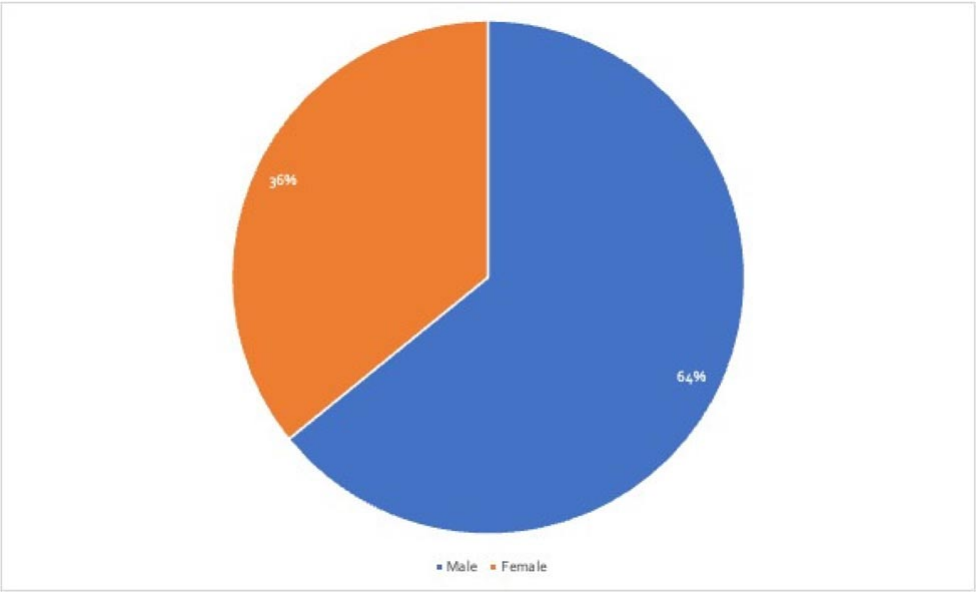
Gender Distribution of Patients Treated with Cefiderocol

**Table 1:**
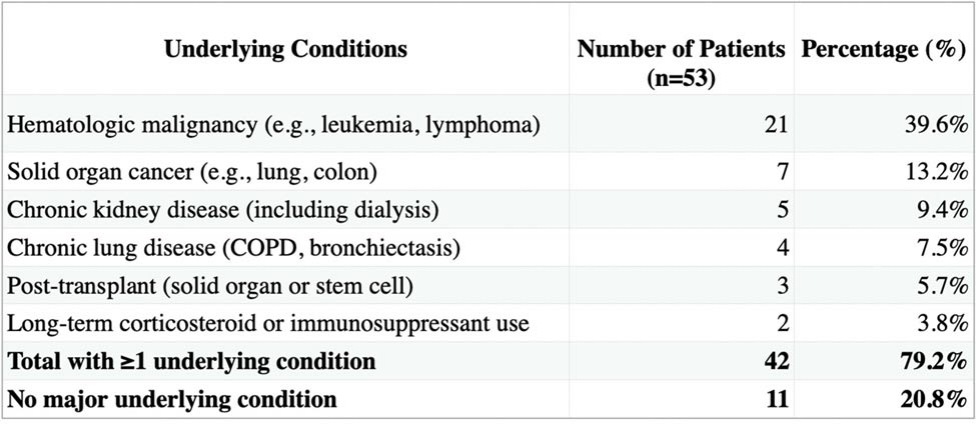
Underlying Conditions in Patients with S. maltophilia Infection

**Results:**

46/53 patients achieved full recovery (Figure 1). Most cases originated from North America (NA) and Europe (EU) 44/53 (Figure 2). A male predominance was noted, comprising 34/53 cases (Figure 3). 42/53 infections developed in patients with comorbidities, with underlying hematologic malignancies being the most common at 39.6% (Table 1). 42/53 of patients were already hospitalized with 30 of them being in the ICU, showing a link between infection and the healthcare setting. The most effective regimen was CFDC combined with TMP-SMX, achieving a 92% recovery rate, followed by CFDC monotherapy at 88%, CFDC plus levofloxacin (Lfx) had the lowest success rate, with the only recorded death (Figure 1). This highlights the importance of selecting optimal combination regimens to improve outcomes in SM infections.

**Conclusion:**

More SM infections were seen in males, and immunocompromised hosts primarily from NA and EU. CFDC, when combined with TMP-SMX, yielded the most favorable outcomes. Monotherapy with CFDC also demonstrated high success rates, whereas combinations with minocycline or Lfx had lower recovery rates. CFDC as a first-line agent against MDR-SM is promising, when used early and in combinations. Further studies are needed to standardize treatment protocols and confirm these findings.

**Disclosures:**

All Authors: No reported disclosures

